# Bioelectrical impedance vector analysis and phase angle to verify early hydration status and prognosis in hospitalized children with nephrotic syndrome: an exploratory case series

**DOI:** 10.3389/fnut.2025.1588452

**Published:** 2025-09-16

**Authors:** Esther Artuanne Figueredo da Silva, Mayara Gabrielly Germano de Araújo, Ana Márcia Soares Fernandes Xavier, Ana Karina da Costa Dantas, Eduardo Paixão da Silva, Márcia Marília Gomes Dantas Lopes

**Affiliations:** ^1^Postgraduate Program in Sciences Applied to Women’s Health, Januário Cicco Maternity School, Federal University of Rio Grande do Norte, Natal, Rio Grande do Norte, Brazil; ^2^Multidisciplinary Residency in Health, Onofre Lopes University Hospital, Federal University of Rio Grande do Norte, Natal, Rio Grande do Norte, Brazil; ^3^Department of Pediatrics, Federal University of Rio Grande do Norte, Natal, Rio Grande do Norte, Brazil; ^4^Postgraduate Program in Public Health, Medicine College, Federal University of Ceará, Fortaleza, Ceará, Brazil; ^5^Postgraduate Program in Sciences Applied to Women’s Health and Department of Nutrition, Federal University of Rio Grande do Norte, Natal, Rio Grande do Norte, Brazil

**Keywords:** nephrotic syndrome, pediatrics, electric impedance, body composition, prognostic factor

## Abstract

**Background:**

Children with nephrotic syndrome (NS) represent a high-risk group for significant clinical and nutritional alterations. The scarcity of studies on rapid and practical methods for assessing hydration status and nutritional prognosis in this context highlights the need for this study.

**Aim:**

To investigate the body composition profile through bioelectrical impedance vector analysis (BIVA) and to evaluate the phase angle (PhA) between groups of hospitalized children with nephrotic syndrome (NS).

**Methods:**

In this study, we present nine cases of hospitalized children diagnosed with NS. The cases were divided into two groups: symptomatic NS (G1) and asymptomatic NS (G2). Upon hospital admission, socioeconomic, clinical, and biochemical data were collected, along with nutritional screening, anthropometric assessment (height-for-age), and body composition analysis using BIVA and PhA calculation.

**Results:**

Most children were male (67%) with a median age of 42 months. Most of the cases received social benefits, and their mothers had completed high school. All patients presented some degree of risk of malnutrition, regardless of symptomatology. BIVA identified anasarca and low body cell mass in the G1 group, whereas the G2 group showed a tendency toward leanness and cachexia. The PhA was significantly lower in group G1 (median = 2.49°, IQR = 1.04) compared to G2 (median = 3.68°, IQR = 0.60) (*p* = 0.036).

**Conclusion:**

BIVA rapidly and early detected extracellular water accumulation and reduced body cell mass, highlighting that those individuals with symptomatic NS had a lower PhA, suggesting a less favorable prognosis.

## 1 Introduction

Nephrotic syndrome (NS) is characterized by intense proteinuria, leading to hypoalbuminemia and edema formation. These symptoms reflect increased glomerular permeability and can lead to malnutrition, poor growth, complications, and longer hospital stays. Although idiopathic NS is the most common glomerular disease in children, its incidence is relatively low, ranging from 1.4 to 6.1 cases per 100,000 children, depending on ethnicity (1).

Furthermore, healthcare professionals often use body weight as an indicator to assess the degree of edema, the disease state, and to guide therapeutic decisions, such as fluid management, dose adjustments, and medication changes. However, this practice is reliable only for short periods, particularly until weight variations attributable to other causes become significant (2).

Therefore, it is essential to identify and adopt more accurate methods that are easy to apply in daily practice, rapid, cost-effective, and non-invasive. Bioelectrical impedance analysis (BIA) fits into this context, as it measures the resistance of different tissues to a small electrical current (3). However, traditional BIA estimates fluid volume using regression equations that can be inaccurate in cases of significant hydration changes, and its body composition estimates require specialized knowledge for a clinical context, especially in non-ideal states. On the other hand, BIVA is superior because it directly assesses hydration status and soft tissue mass without relying on body weight or regression equations. This makes it more accurate in clinical conditions with fluid imbalance. BIVA is sensitive to subtle changes in hydration, which is crucial in diseases where precise fluid volume control is essential to avoid complications. Furthermore, its graphical representation (vectors in tolerance nomograms) allows for a visual and immediate assessment of hydration status and cell mass, facilitating interpretation and treatment monitoring in complex clinical scenarios (4–8).

The ability of BIVA to predict clinical outcomes in children with complex diagnoses has been described in the literature, and it has also been identified as an alternative technique to assess hydration status in various clinical situations (7–10).

Additionally, R and Xc can provide phase angle (PhA) values, a simultaneous marker of cell mass and health, recognized as a reliable prognostic marker, including those involving the pediatric population (11–14). PhA reflects changes in the body’s electrical conductivity, highlighting alterations in cell membrane integrity and intercellular space (15).

The use of precise parameters to monitor the clinical and nutritional status of children with NS has been widely discussed in the literature. Brantlov et al. demonstrated that PhA and BIVA are effective in distinguishing children with NS from healthy controls and are reliable tools for monitoring disease status (8). These findings underscore the importance of further research into parameters that can inform nutritional prognosis and support clinical decision-making (16). Currently, studies using BIVA to assess pediatric patients with NS remain limited (7, 8).

Considering that fluid distribution is a characteristic of NS, edema typically becomes clinically detectable only when interstitial fluid increases by at least 30% above normal. Accordingly, BIVA emerges as a non-invasive, cost-effective strategy for monitoring nutritional status when actual body weight measurement is unreliable. There are no studies on the use of BIVA and PhA comparing symptomatic and asymptomatic patients during hospitalization. Thus, in this report, we present an exploratory case series of children hospitalized with NS, using BIVA to identify differences in body composition between symptomatic and asymptomatic patients, describing demographic, clinical, and nutritional factors, and conducting a discussion to gain a better understanding of the use of BIVA and PhA in children with NS.

## 2 Materials and methods

### 2.1 Population and study design

This exploratory case series included children and adolescents with NS who were hospitalized in the pediatric ward and diagnosed based on the Kidney Disease Outcomes Quality Initiative (KDOQI) criteria (17), between June and October 2021. The study included cases aged 1–15 years (12–180 months). The exclusion criteria were diagnosis of liver disease, endocrine disorders, creatinine clearance <60 mL/min/1.73 m^2^, and those in palliative care. This study was approved by the Research Ethics Committee of the Onofre Lopes University Hospital (number 4.623.568), with informed consent obtained from the guardians and assent from participants over 6 years of age.

### 2.2 Clinical data collection

Data collected from patients’ physical and electronic medical records included personal information (date of birth and sex), clinical data (age at diagnosis, hospitalization with symptomatic or asymptomatic NS, prior and current medication and supplement use), biochemical data (lipid profile, urea, albumin, sodium, potassium, and C-reactive protein—CRP-), and socioeconomic data (family income, receipt of benefits, and maternal education). Clinical findings of edema were assessed by a pediatric nephrologist based on physical examination, including signs such as periorbital swelling, lower limb edema, ascites, and pitting edema. Additionally, nutritional information was recorded, including nutritional screening, height measurement, and BIA assessment at hospital admission.

### 2.3 Biochemical assessment

Blood collection was performed in the morning after a 12-h fast, using venipuncture with sterile, disposable plastic syringes and stainless-steel needles. The quantification of biochemical parameters was conducted using specific methods. Urea was measured by the kinetic UV method. Sodium and potassium levels were measured using the potentiometric method with ion-selective electrodes (ISEs), while C-reactive protein (CRP) was determined by immunoturbidimetry. Colorimetric enzymatic assays were used to analyze albumin, total cholesterol, and triglycerides. High-density lipoprotein cholesterol (HDL-c) was measured using a specific colorimetric method. Low-density lipoprotein cholesterol (LDL-c) was calculated using Friedewald’s formula: LDL-c = (total cholesterol – HDL-c) – (triglycerides/5), which is valid only when triglyceride levels are below 400 mg/dL. All analyses were conducted in the hospital’s clinical laboratory using the CMD 800i X1 chemical analyzer (Diamond Diagnostics®, Holliston, MA, USA) and reagent kits from Wiener Lab® (Wiener® Lab Group, Argentina).

### 2.4 Nutritional and body composition assessment

Upon admission, a nutritional risk screening was conducted using the Strong Kids tool. Additionally, height was measured, and BIA was performed, providing values for R and Xc. Trained registered dietitians performed these procedures. The nutritional risk level was determined based on the score from the Strong Kids tool. Children with a score of 0 were classified as low risk, scores of 1–3 indicated moderate risk, while scores of 4–5 identified those at high risk for malnutrition (18).

Height was measured using a stadiometer (Professional Sanny, American Medical do Brazil, São Paulo, SP, Brazil) following the protocol established by Warrier et al. (19). The height-for-age z-scores were calculated using the Anthro and Anthro Plus software and classified according to the World Health Organization (WHO) growth curves for healthy children and adolescents (20, 21).

For the bioelectrical assessment, the Quantum II® impedance analyzer (RJL Systems, Clinton Township, MI, USA) was used to obtain the values for R (Ω) and Xc (Ω) measured at 50 kHz. The measurement was performed with the participant lying in a supine position, with four electrodes placed—two on the dorsal surface of the right hand and two on the dorsal surface of the right foot, as described by Lukaski et al. (22). Each participant was instructed to fast before the measurement, which was conducted around 7 a.m., and to empty their bladder. The nursing staff reinforced these instructions and verbally confirmed adherence with the guardian or patient before the assessment.

The R and Xc values were used for BIVA and PhA calculation. For BIVA, a graphical method of R and Xc was corrected by the individual’s height in meters. Bivariate vectors were plotted on the RXc graph using the BIVA 2002 software. Individual measurements were compared to tolerance ellipses derived from a healthy population and calculated using BIVA software (23). These ellipses represent the 50, 75, and 95% tolerance intervals of reference values previously established in an Italian population by De Palo, indicating on the graph the individual’s nutritional status regarding cellularity and body hydration. An individual considered ideally healthy, that is, with a complete balance in the number of cells and body water and cellular integrity, has their vector positioned at the center of the 50% ellipse. Any deviation outside the 50 and 75% ellipses, in any direction on the graph (above, below, left, or right), where the vectors are positioned within the 95% and above 95% ellipses, demonstrates an imbalance in cellular homeostasis, whether due to diseases that lead to cachexia, dehydration, or overhydration, or due to obesity or significant muscle mass gain (24). PhA, calculated using the formula arctangent (Xc/R) × (180/*π*), is an indicator of cell membrane integrity and the proportion of intact cells. Higher PhA values generally indicate better cell mass and function and are associated with a better prognosis in various clinical conditions (25).

Participants were grouped by age and sex and further categorized into Group 1 (G1) and Group 2 (G2) based on disease status at admission. G1 included children with symptomatic NS, defined by the presence of generalized edema (anasarca) assessed by a pediatric nephrologist, hypoalbuminemia (serum albumin <2.5 g/dL), and elevated urinary protein levels. G2 included those with asymptomatic disease, without edema, and elevated urinary protein levels.

### 2.5 Statistical analysis

For the statistical analysis, the data distribution was assessed by calculating the median for non-normally distributed data. Data normality was checked using the Shapiro–Wilk test, while other variables were analyzed based on relative frequencies. The dataset was checked for missing and outlier values. No missing data were identified for the variables assessed. Outliers were assessed using boxplots and clinical plausibility; as none were deemed erroneous or biologically implausible, all values were retained for analysis.

The independent samples t-test (Mann–Whitney U test) was used to analyze the statistical difference between continuous variables in groups G1 and G2. Fisher’s exact test was used when comparing proportions between two nominal variables, each with two categories. Statistical analyses were performed using JASP software (JASP Team, 2024), JASP (Version 0.19.3 software), and BIVA 2002® software (Microsoft, Padova, Italy). The BIVA evaluation of participants relied on the reference population described by De Palo et al. (26).

## 3 Results

Of the nine cases, 67% were male, and the main cause of hospitalization was symptomatic disease (55.6%).

Regardless of the presence of symptoms, most children received social benefits, and their mothers had completed high school, and all patients presented some degree of risk of malnutrition. Furthermore, patients with symptomatic disease spend more days in the hospital (*p* = 0.011, Cramer’s V = 1.000) (Table 1).

**Table 1:** Socioeconomic, clinical, and nutritional characteristics of hospitalized children with NS according to symptom presence.

Variables	Group 1 (Symptomatic) (*n* = 6)	Group 2 (Asymptomatic) (*n* = 3)	*p* value
Age at diagnosis, median (IQR*) in months^1^	21.0 (48.0)	38.0 (4.3)	= 0.070
Current age, median (IQR*) in months^1^	42.0 (46.0)	54.0 (43.0)	= 0.300
Family income^2^, %			= 0.276
Up to 1 Minimum Wage**	83.3%	33.3%	
1–2 Minimum Wages**	16.7%	66.7%	
Receipt of social benefits^2^, %			= 0.343
Yes	33.3%	33.3%	
No	66.7%	66.7%	
Maternal education^2^, %			= 0.453
Incomplete Elementary Education	16.7%	33.3%	
Complete Elementary Education	16.7%	–	
Complete High School	50%	66.7%	
Complete Higher Education	16.7%	–	
Nutritional risk^2^, %			= 0.453
Medium	83.3%	100%	
High	16.7%	–	
Height-for-age z score classification^2^, %			= 0.453
Low	16.7%	–	
Adequate	83.3%	100%	
Hospitalization length^2^, %			**= 0.011**
≤5 days	–	100%	
Between 6 and 10 days	50%	–	
≥10 days	50%	–	
Routine medication^2^, %			
Corticosteroid	66.7%	100%	= 0.257
Immunosuppressant	33.3%	33.3%	= 1.000
Antihypertensive	100%	66.7%	= 0.134
Vitamin D supplementation	50%	–	= 0.134

*IQR: Interquartile range; **Minimum wage (value in 2020: R$ 1,100.00).

^1^Mann–Whitney U test; ^2^Fisher’s Exact Test. Statistical significance values are given in bold.

**Figure 1:**
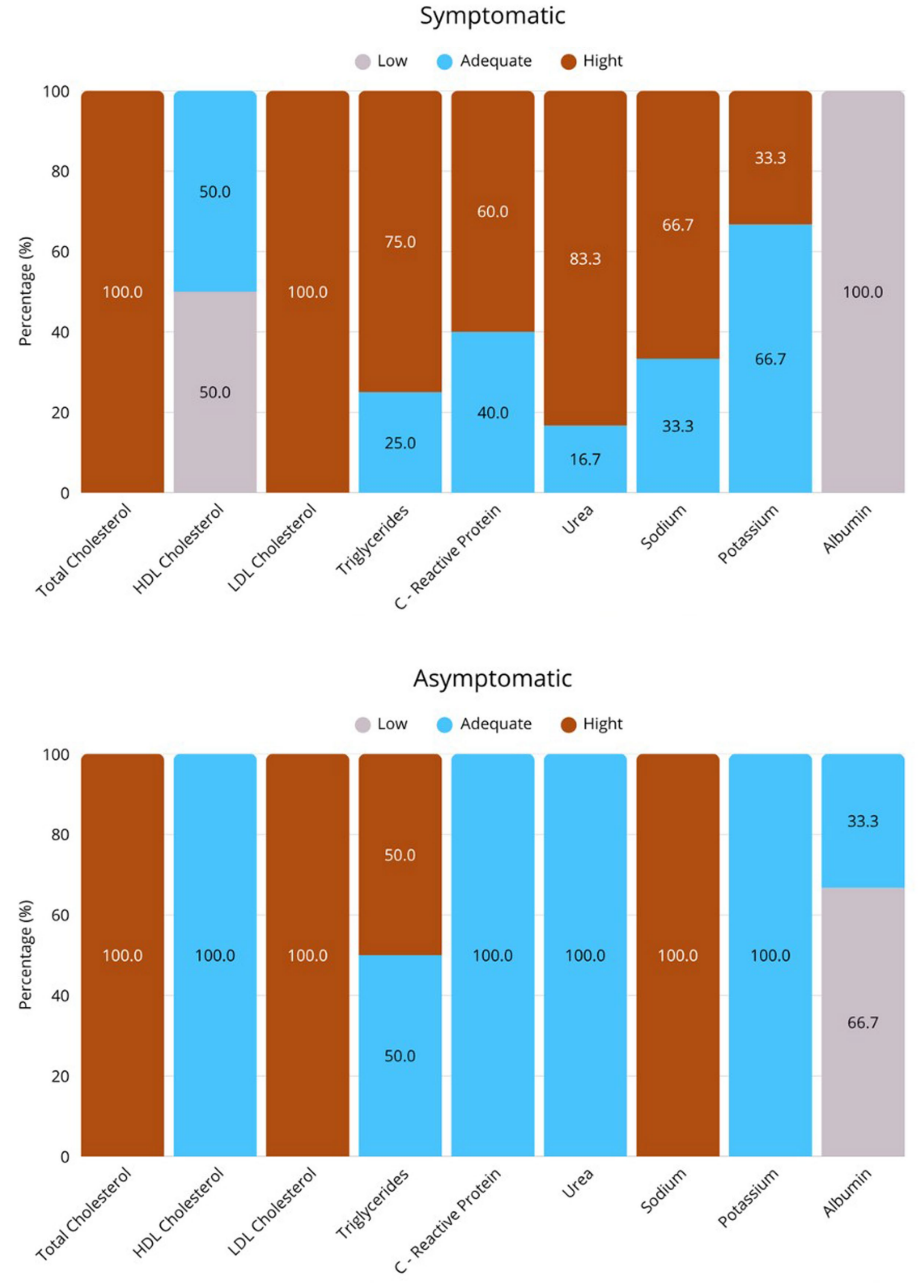
Descriptive biochemical profile of study participants. **(A)** Group of asymptomatic NS, and **(B)** Group of symptomatic NS. Values expressed in %. TC, total cholesterol, LDL, low-density lipoprotein, HDL, high-density lipoprotein, TAG, triglycerides, CRP, C-reactive protein.

Figure 1 presents the descriptive biochemical profile of the patients at admission. We observed that, regardless of symptomatic disease stage, all patients exhibited elevated total cholesterol and LDL levels, while most participants showed increased sodium and triglyceride levels. We found that the majority of patients with symptomatic disease had elevated urea, CRP, and potassium. For albumin, the values were adequate only in a small proportion of asymptomatic patients.

**Figure 2:**
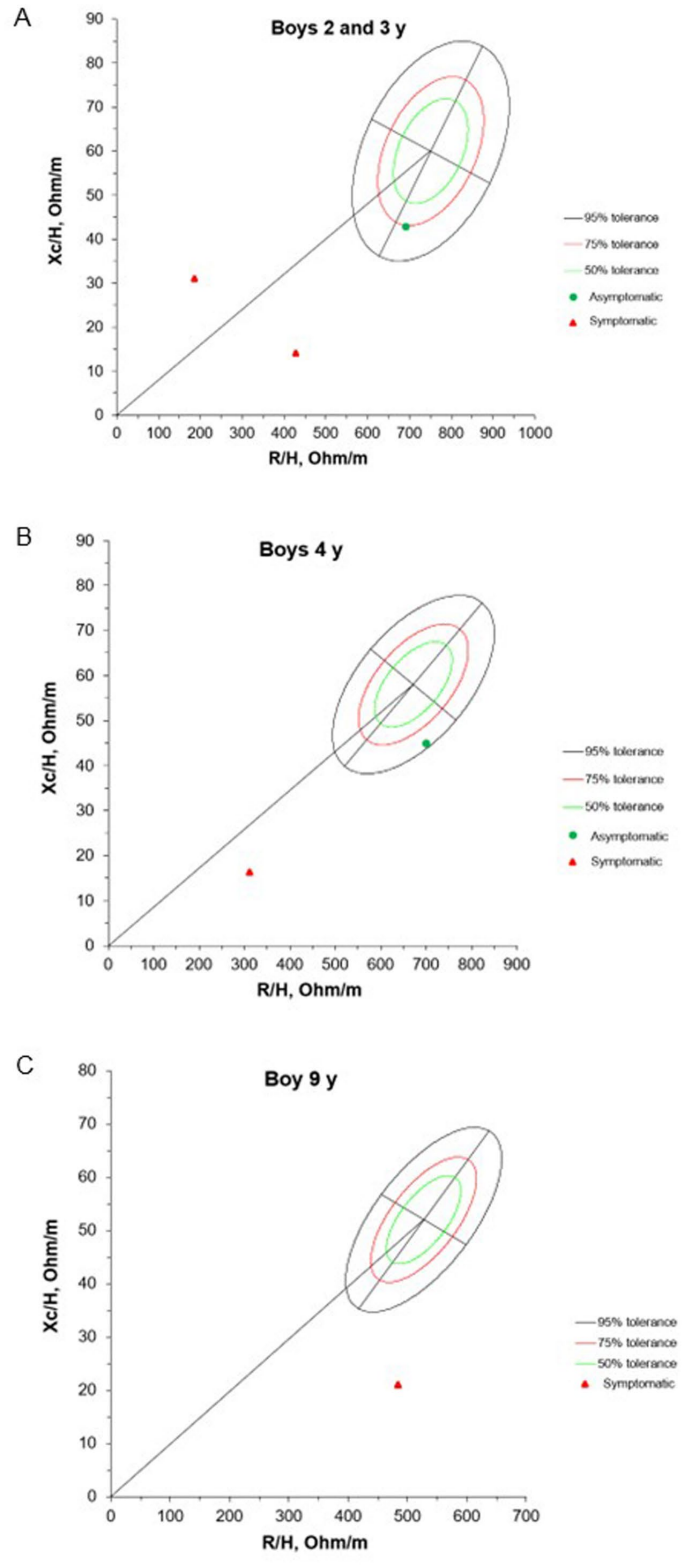
Mean impedance vectors with tolerance ellipses of 50%, 75% and 95% of boys asymptomatic and symptomatic at the hospital admission, according to age. Xc/H: Reactance/height, R/H: Resistance/height.

**Figure 3:**
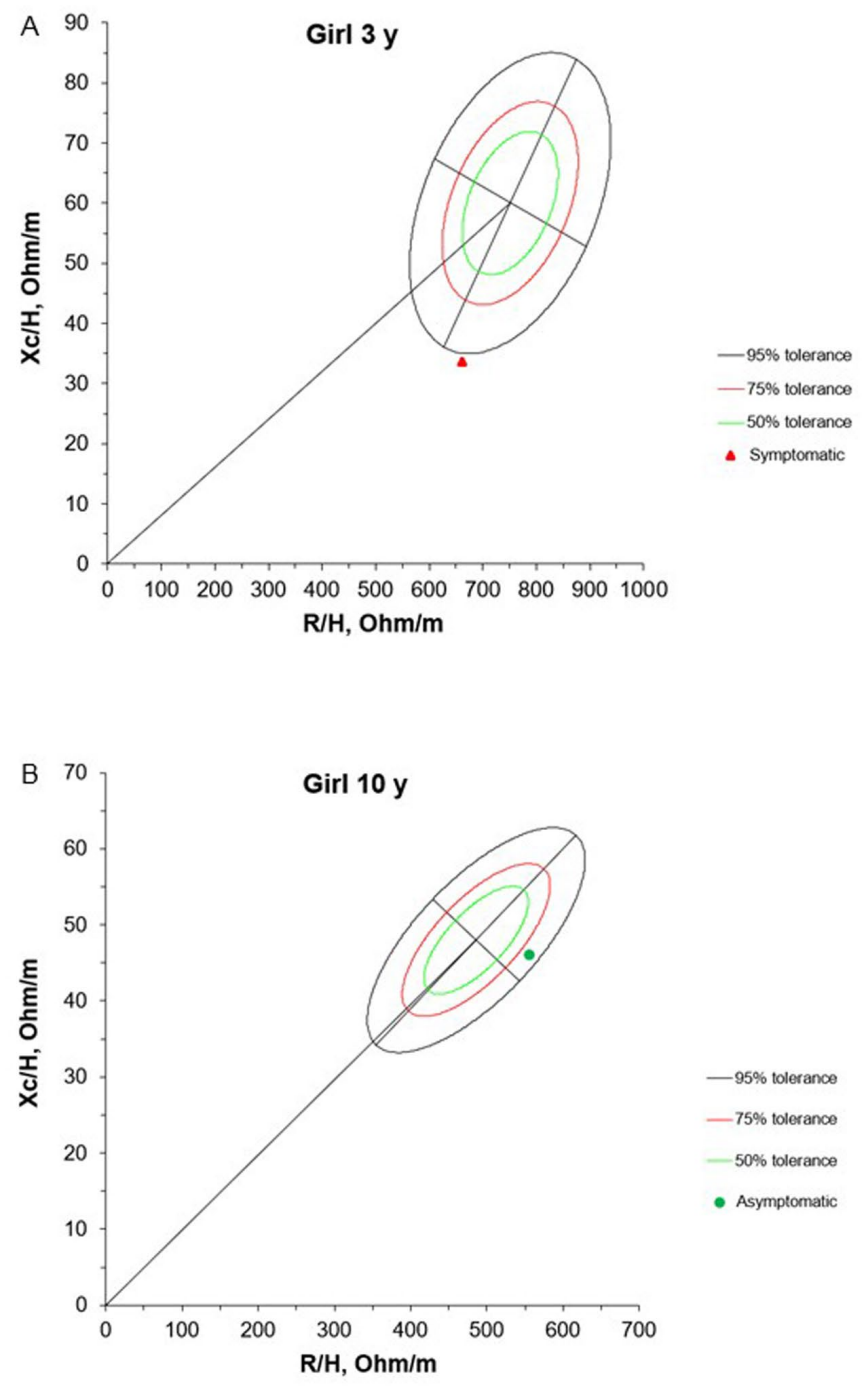
Mean impedance vectors with tolerance ellipses of 50%, 75% and 95% of girls asymptomatic and symptomatic at the hospital admission, according to age. Xc/H: Reactance/height, R/H: Resistance/height. * A 1-year-old girl was excluded from this evaluation due to the lack of a reference population for comparison purposes in this age group.

The BIVA results at admission for children with NS are presented in Figures 2, 3. Due to the lack of an appropriate reference population for this age group, the 1-year-old infant was excluded from the evaluation. Among the 8 individuals evaluated, 62.5% had vectors outside the tolerance ellipses, indicating anasarca. These patients belong to G1 and are identified by red dots in the figure. On the other hand, participants in G2, represented by green dots, showed vectors within the 75 and 95% ellipses, indicating a tendency toward low cell mass and cachexia.

**Figure 4:**
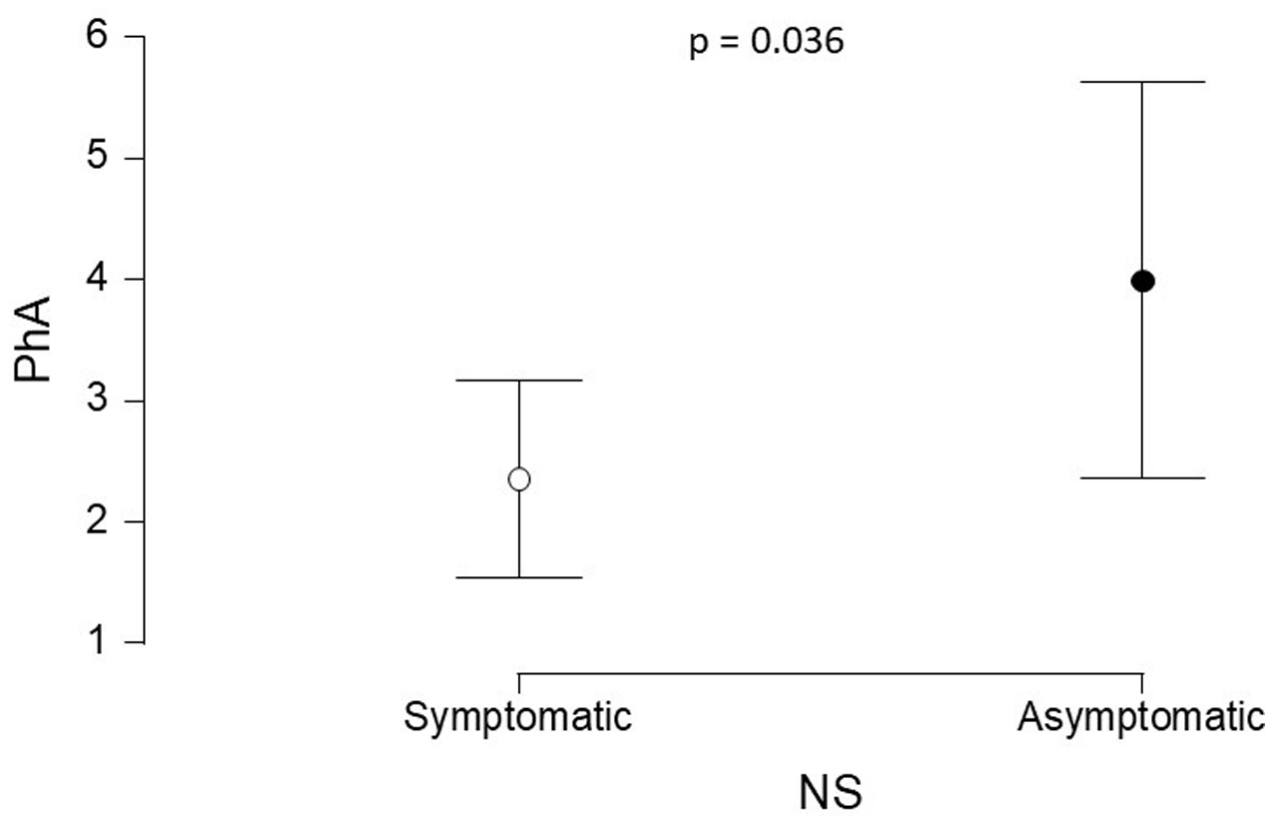
Comparison of the PhA values between G1 and G2. PhA: phase angle, G1 - patients with symptomatic NS (95% CI: 1.541, 3.167); G2 - patients with asymptomatic NS (95% CI: 2.358, 5.629).

The PhA was significantly lower in group G1 (median = 2.49°, IQR = 1.04) compared to G2 (median = 3.68°, IQR = 0.60) (*p* = 0.036) (Figure 4).

## 4 Discussion

This exploratory case series, conducted in Brazil, is pioneering in its analysis of body composition parameters using BIVA and in describing demographic, clinical, and nutritional characteristics of hospitalized children with NS. Our results reveal that all children exhibited some degree of nutritional risk, with a high prevalence of mixed dyslipidemia and proteinuria, common in children with NS. The BIVA assessment revealed distinct patterns between the patient groups. Most participants showed vectors outside the tolerance ellipses, indicating complexities in both hydration status and body composition. These findings are noteworthy and underscore the importance of an integrated and personalized approach to disease management, one that considers the multiple dimensions of health and wellbeing.

Furthermore, our findings support the association between low income and increased risk of chronic conditions such as NS (27, 28). Additionally, limited maternal education (44.4% completed high school) may affect access to health information and disease management, contributing to greater vulnerability to complications (29–31).

Previous studies show a higher prevalence of NS in males, a pattern also observed in our sample (32, 33). The median age at diagnosis was 24 months (IQR 11), with those hospitalized for symptomatic disease at younger ages, consistent with León et al. and Lage, who reported 24–48 months as the most common diagnostic age (34, 35). These findings highlight the importance of early diagnosis and close monitoring in young children to ensure timely treatment. Although growth deficiency is common in NS, only a minority of our patients exhibited short stature, possibly because some were recently diagnosed. Bellot et al. found similar results, with 18.2% of hospitalized children showing short stature (36).

All children and adolescents in the study exhibited some degree of nutritional risk, predominantly moderate, consistent with literature highlighting malnutrition in NS resulting from protein loss and metabolic alterations (37). This variability likely reflects disease severity and treatment response, as longer hospital stays are often linked to more severe or complicated cases (38). Bellot et al. supported this view (36), revealing a 100% nutritional risk in their sample of children with NS, using the same screening tool. Nutritional screening, as recommended by the Brazilian Association of Nutrition, is essential for the early identification of at-risk patients, enabling timely and appropriate nutritional interventions. These findings emphasize the importance of a systematic approach in nutritional care, aiming to improve both the management and prognosis of these patients (39, 40).

The biochemical profile of the patients revealed mixed dyslipidemia, hypoalbuminemia, and hypernatremia. These findings are characteristic of NS and align with previous studies that have documented these biochemical imbalances as typical manifestations of the disease (41, 42). The elevation of CRP in half of the patients, especially in G1, indicates active inflammation, which may be associated with an exacerbated inflammatory response in NS (43).

We also observed changes in urea and potassium in a minority of patients, with these changes associated with the presence of infection. In line with our findings, Rheault et al. identified that 6% of a sample of 336 children hospitalized for NS showed evidence of acute kidney injury, with concomitant infection, the use of nephrotoxic medications, and steroid-resistant NS being risk factors (44).

Additionally, Noone et al. emphasize that steroid-resistant NS is associated with a high risk of progression to end-stage renal disease (45). Although all patients were diagnosed with mixed dyslipidemia, the use of lipid-lowering medications was observed in only a minority. This pattern can be explained by the recommendation that lipid-lowering agents should not be used in NS unless there is persistent proteinuria with extremely elevated triglyceride levels (46).

In hospital management of edema, various medications are employed, including loop diuretics, thiazide diuretics, and thiazide-like agents, with furosemide being the most commonly used (47). Edema is widely recognized as one of the primary symptoms of NS and remains one of the leading reasons for hospitalization in these patients (48). However, accurate assessment of intravascular volume is challenged by the lack of an established gold standard for this measurement (8). In this context, BIVA has emerged as a practical tool for analyzing body fluids, offering advantages such as ease of application, being noninvasive, and good reproducibility (10).

BIVA patterns are based solely on the electrical properties of tissues, without considering body weight. BIVA can detect changes in hydration and tissue structure, as both R and Xc are considered simultaneously. Research has demonstrated that BIVA is an excellent tool for identifying individual vectors, detecting changes in tissue hydration and body composition, and serving as a powerful method for evaluation and monitoring (49–52).

Our results showed that 62.5% of patients had vectors outside the tolerance ellipses, indicating anasarca and fluid overload in symptomatic NS. These findings align with the pathophysiology of NS, where hypoalbuminemia decreases oncotic pressure, leading to extravascular fluid accumulation (51). In contrast, G2 patients had vectors within the 75 and 95% ellipses, suggesting lower body cell mass and possible muscle wasting. This pattern highlights the importance of tailored nutritional and fluid management. Integrating BIVA and PhA into hospital care can enhance early detection of hydration and nutritional imbalances often overlooked by routine assessments, thereby guiding timely interventions—such as diuretic therapy and nutritional support—to improve patient outcomes.

The significant difference in PhA between groups G1 and G2 reflects greater fluid overload and cellular impairment in patients with symptomatic NS (G1) compared to those with asymptomatic NS (G2). PhA, a recognized indicator of cell membrane integrity and body cell mass, decreases in response to cellular damage and malnutrition (52). This parameter can detect subclinical changes in cellular health before they manifest as anthropometric deficits, thus serving as an early and sensitive marker of nutritional risk (53). Incorporating PhA monitoring into clinical practice allows for earlier identification of patients at higher risk of complications, enabling timely nutritional and fluid management interventions that can improve clinical outcomes and individualize care in pediatric NS.

For healthy individuals, the PhA value typically ranges between 5 and 19 (24). According to a study conducted by De Palo et al. with 2,044 healthy children aged 10–15 years, the variation of PhA was between 5.7° and 6.2° (26). Our results, regardless of the presence of decompensation, showed PhA values below these reference standards. These findings suggest a potential alteration in cellular membrane integrity and the intercellular space, indicating compromised cellular health and function.

To our knowledge, this exploratory case series is the first to investigate BIVA and PhA in hospitalized children to detect body composition changes as early as the first day of admission. Although the small sample size limits broader generalizability, the internal consistency of the results underscores the exploratory significance of the study. These findings highlight the need for future studies with larger, longitudinal cohorts to validate and extend these observations. Still, our results align with prior research on BIVA in pediatric NS (7, 8).

Comparisons between BIVA and other hydration assessment methods in this population are also warranted. Despite these limitations, our study underscores the potential of body composition analysis to enhance clinical and nutritional management in hospitalized pediatric patients with NS. BIVA provides a detailed, noninvasive tool to assess fluid status, while PhA reflects cell membrane integrity and nutritional condition, supporting its use as a potential biomarker in this context. Given the characteristic edema and fluid distribution alterations in NS, BIVA may help differentiate fluid overload from true changes in body composition, contributing to more precise clinical decision-making. Future research should involve larger cohorts and longitudinal designs to track PhA dynamics throughout treatment and recovery.

Despite the small sample size, our findings suggest that integrating BIVA and PhA yields clinically consistent data. Key contributions of this case series include: (1) while all patients were at moderate-to-high nutritional risk by conventional tools, only BIVA and PhA clearly distinguished the two groups in terms of nutritional status and fluid overload; (2) both methods proved to be practical, rapid, and noninvasive, supporting ongoing assessment in challenging clinical scenarios such as anasarca; (3) the associations observed among hydration, body composition, and PhA have direct clinical relevance for managing pediatric NS; and (4) in symptomatic children, bioelectrical vector analysis indicated excess extracellular fluid and reduced cell mass, findings often masked by edema in standard assessments. The adoption of these methods in hospital routines can favor more individualized, early, and targeted therapeutic decision-making before the clinical condition visibly worsens, in addition to enabling more precise monitoring of the response to nutritional and pharmacological treatment.

## 5 Conclusion

In conclusion, according to this case series, BIVA revealed, upon admission, an accumulation of extracellular water and a reduction in cell mass, which were more pronounced in patients with symptomatic NS and lower lean body mass, while PhA indicated a poorer prognosis. The study demonstrates that BIVA and PhA are useful tools to complement clinical and biochemical assessment, offering a more integrated and dynamic view of the patient’s condition. These methods have proven useful for routine interventions, as they allow for the rapid, practical, and early detection of relevant alterations that influence the prognosis of NS.

Finally, further studies with larger sample sizes are essential to validate and build upon these findings. Specifically, we suggest that BIVA be used as an adjunctive assessment, used in conjunction with a comprehensive multidisciplinary approach.

## Data Availability

The raw data supporting the conclusions of this article will be made available by the authors without undue reservation.
